# Therapeutic Efficacy of Excretory-Secretory Products of *Trichinella spiralis* Adult Worms on Sepsis-Induced Acute Lung Injury in a Mouse Model

**DOI:** 10.3389/fcimb.2021.653843

**Published:** 2021-03-24

**Authors:** Huihui Li, Dapeng Qiu, Huijuan Yang, Yuan Yuan, Lingqin Wu, Liang Chu, Bin Zhan, Xiaoli Wang, Yan Sun, Wei Xu, Xiaodi Yang

**Affiliations:** ^1^ Department of Basic Medical College, Bengbu Medical College, Bengbu, China; ^2^ Anhui Key Laboratory of Infection and Immunity of Bengbu Medical College, Bengbu, China; ^3^ Department of Orthopedics, Second Affiliated Hospital of Bengbu Medical College, Bengbu, China; ^4^ National School of Tropical Medicine, Baylor College of Medicine, Houston, TX, United States

**Keywords:** excretory-secretory products, *Trichinella spiralis*, sepsis, acute lung injury, cecal ligation and puncture, immunomodulation

## Abstract

Acute lung injury (ALI) is a common complication of systemic inflammation or sepsis with high morbidity and mortality. Although many studies have confirmed that helminth-derived proteins had strong immunomodulatory functions and could be used to treat inflammatory diseases, there is no report on the therapeutic effect of excretory-secretory products of *Trichinella spiralis* adult worms (*Ts*-AES) on sepsis-induced ALI. In this study, the therapeutic efficacy of *Ts*-AES on sepsis-induced ALI and the underlying immunological mechanism and the signaling pathway were investigated. The results indicated that after being treated with *Ts*-AES, the survival rate of mice with CLP-induced sepsis was significantly increased to 50% for 72 hours after CLP surgery compared to PBS control group with all mice died. The sepsis-induced ALI was largely mitigated characterized by reduced inflammation cell infiltration and pathological changes in lung tissue, with decreased lung injury scores and lung wet/dry weight ratio. The therapeutic efficacy of *Ts*-AES is associated with stimulated Tregs response with increased regulatory cytokines IL-10 and TGF-β and downregulated pro-inflammatory cytokines (TNF-α, IL-6, IL-1β). The expression of HMGB1, TLR2 and MyD88 in lung tissue was inhibited after treatment of *Ts*-AES. Our results demonstrated that *Ts*-AES play an important role in immunomodulation and confer a therapeutic effect on sepsis-induced ALI through inhibiting pro-inflammatory cytokines. The activation of Tregs and increased level of regulatory cytokines IL-10 and TGF-β are possibly involved in the immunomodulatory functions of *Ts*-AES through HMGB1/TLR2/MyD88 signal pathway. The findings suggest *Ts*-AES is a potential therapeutic agent for prevention and treatment of sepsis-induced ALI and other inflammatory diseases.

## Introduction

Sepsis is life-threatening organ dysfunction caused by a dysregulated severe host immune response to an infection (Singer et al., 2016). It can lead to multiple organ injuries and even death affecting the lives of millions of people around the world (Butt et al., 2016; Novosad et al., 2016; Rello et al., 2017). Acute lung injury (ALI) occurs earliest in sepsis and is a main cause of death (Rubenfeld et al., 2005; Rudd et al., 2020). Although there are various therapeutic strategies for ALI which include the administration of nitric ox surfactant and glucocorticoids, none of them reduces the mortality in sepsis-induced ALI (Li et al., 2020). Thus, searching for novel and more effective therapeutic approaches is an urgent need for the treatment of sepsis-induced ALI and reducing the ALI-caused death.

High mobility group box 1 protein (HMGB1), a highly conserved nuclear DNA−binding protein, is a late inflammatory cytokine that activates macrophages and dendritic cells to produce inflammatory cytokines, playing a key role in triggering the inflammatory response in the pathogenesis of ALI and sepsis (Deng et al., 2018; Li and Lu, 2021; Xie et al., 2021). During sepsis, HMGB1 is secreted into the extracellular milieu after stimulating by bacterial endotoxin or pro-inflammatory cytokines. TLRs (TLR2 and TLR4) are reportedly the primary receptors of HMGB1 that transmit intracellular signals by Myd88 to stimulate the secretion of pro-inflammatory cytokines, which in turn promote HMGB1 secretion as a positive feedback loop during the inflammatory process (Lee et al., 2014). It has been shown that HMGB1 was elevated in the sera of septic patients, suppressing HMGB1 levels by pharmacological intervention and reducing CLP-induced sepsis mortality (Rosas-Ballina et al., 2009; Valdés-Ferrer et al., 2013; Deng et al., 2019; Wang J. et al., 2020). These findings suggest that HMGB1 could be a therapeutic target for preventing inflammation and ALI in sepsis.

Since the hygiene hypothesis was proposed by Strachan in 1989 (Strachan, 1989), an increasing number of experiments and epidemiological studies have revealed the inverse correlation between the inflammation, autoimmune or allergic diseases with helminth infections (van Riet et al., 2007; Zaccone et al., 2008; Erb, 2009; Osada and Kanazawa, 2010; Smallwood et al., 2017). Parasitic helminths co-evolve with mammalian hosts and develop some strategies such as activating host Th2-dominant immune response and regulatory T cell response to survive within hosts (O’Regan et al., 2014; Passos et al., 2017; Hernández-Ancheyta et al., 2018). Further evidences have showed that helminths regulated the local or systemic immune response of host through secreting some biofunctional proteins in excretory-secretory (ES) products. For instance, the ES products of *Marshallagia marshalli* had anti-inflammatory potentials on allergic airway inflammation in mice (Shirvan et al., 2016), and ES products of *Brugia malayi* attenuated development of streptozotocin-induced type 1 diabetes in mice associated with the decreased production of pro-inflammatory cytokines and increased production of IL-10 (Amdare et al., 2015).

*Trichinella spiralis* is one of the most widespread zoonotic parasitic nematodes in the world. Its life cycle is completed within a single host including three stages: muscle larvae inside skeletal striated muscle cells, adult worms in the small intestine, and newborn larvae in the lymphatic vessels and bloodstream. During the chronic phase of the *T. spiralis* infection, the adult worms or muscle larvae secrete or release ES products into host to activate regulatory network elements to reduce host immune attack as a survival strategy (Bai et al., 2012; Sofronic-Milosavljevic et al., 2015; Kosanović et al., 2019). ES products derived from different developmental stages of *T. spiralis* may act different immunomodulatory effects (Yang et al., 2014; Jin et al., 2019a). Many experimental studies have shown that infection with *T. spiralis* or exposure to ES products of *T. spiralis* induced a strong Th2/Treg response correlated with the stimulation of anti-inflammatory cytokines (IL-4, IL-5, and IL-13) and regulatory cytokines (IL-10, TGF-β), as well as the inhibition of pro-inflammatory cytokines (TNF-α, IL-6 and IL-1β) (Han et al., 2019; Sun et al., 2019b; Ding et al., 2020). *T. spiralis* ES has been successfully used for the treatment of inflammatory bowel diseases (Yang et al., 2014; Jin et al., 2019a; Wang Z. et al., 2020), allergic asthma (Sun et al., 2019a) and sepsis (Du et al., 2014) in mouse models.

In this study, we explored the therapeutic effect of ES products from *T. spiralis* adult worms (*Ts*-AES) on CLP-induced septic ALI in BALB/c mice and we found that treatment with *Ts*-AES significantly improved the survival rate against systematic sepsis and reduced the sepsis-induced ALI in mouse model, providing a potential treatment method to control sepsis-induced ALI and death.

## Materials and Methods

### Animals

Male specific pathogen-free BALB/c mice (6-8 weeks old with weight of 18-22 g), female ICR mice (6-8 weeks old with weight of 25-30 g) and female Wistar mice (6 weeks old with weight of 180-220 g) were purchased from the Animal Center of Bengbu Medical College and maintained under controlled temperature of 20-25°C, relative humidity of 40-55% and a 12:12 h light/dark cycle. All animals received a normal rodent diet and free access to water. All experiments were performed in accordance with protocols approved by Animal Care and Use Committee of Bengbu Medical College (approval no: LAEC-2014-039).

### Preparation of *Ts*-AES

*T. spiralis* muscle larvae were isolated from the muscles of infected female ICR mice by a previously described method of modified pepsin-hydrochloric acid digestion (Gu et al., 2013) as a source for infection. Each Wistar rat was orally infected with 12,000 muscle larvae of *T. spiralis* (Martínez-Gómez et al., 2009; Sun et al., 2019b) and the adult worms were collected from intestines of mice 106 hours post infection. The collected adult worms were washed with phosphate-buffered saline (PBS) for three times and then cultured in RPMI-1640 medium (Hyclone, Logan, UT, USA) supplemented with 100 U/mL penicillin and 100 µg/mL streptomycin (Gibco, Grand Island, NY, USA) at 37°C, 5% CO_2_ for 48 h. The culture supernatant containing *Ts*-AES was collected and concentrated by centrifugating and buffer exchanged into PBS. The potential contaminated endotoxin in *Ts*-AES products was removed using a ToxOut™ High Capacity Endotoxin Removal Kit (BioVision, Palo Alto, California, USA) and confirmed using ToxinSensor™ Chromogenic Limulus Amebocyte Lysate (LAL) Endotoxin Assay Kit (GenScript Biotechnology, Nanjing, China) following the manufacturer’s protocol. The protein concentration of the prepared *Ts*-AES was determined by Bicinchoninic Acid Protein Assay Kit (Beyotime Biotechnology, Shanghai, China) and then stored at -80°C until use.

### Sepsis Induced by Cecal Ligation and Puncture (CLP)

**Figure 1:**
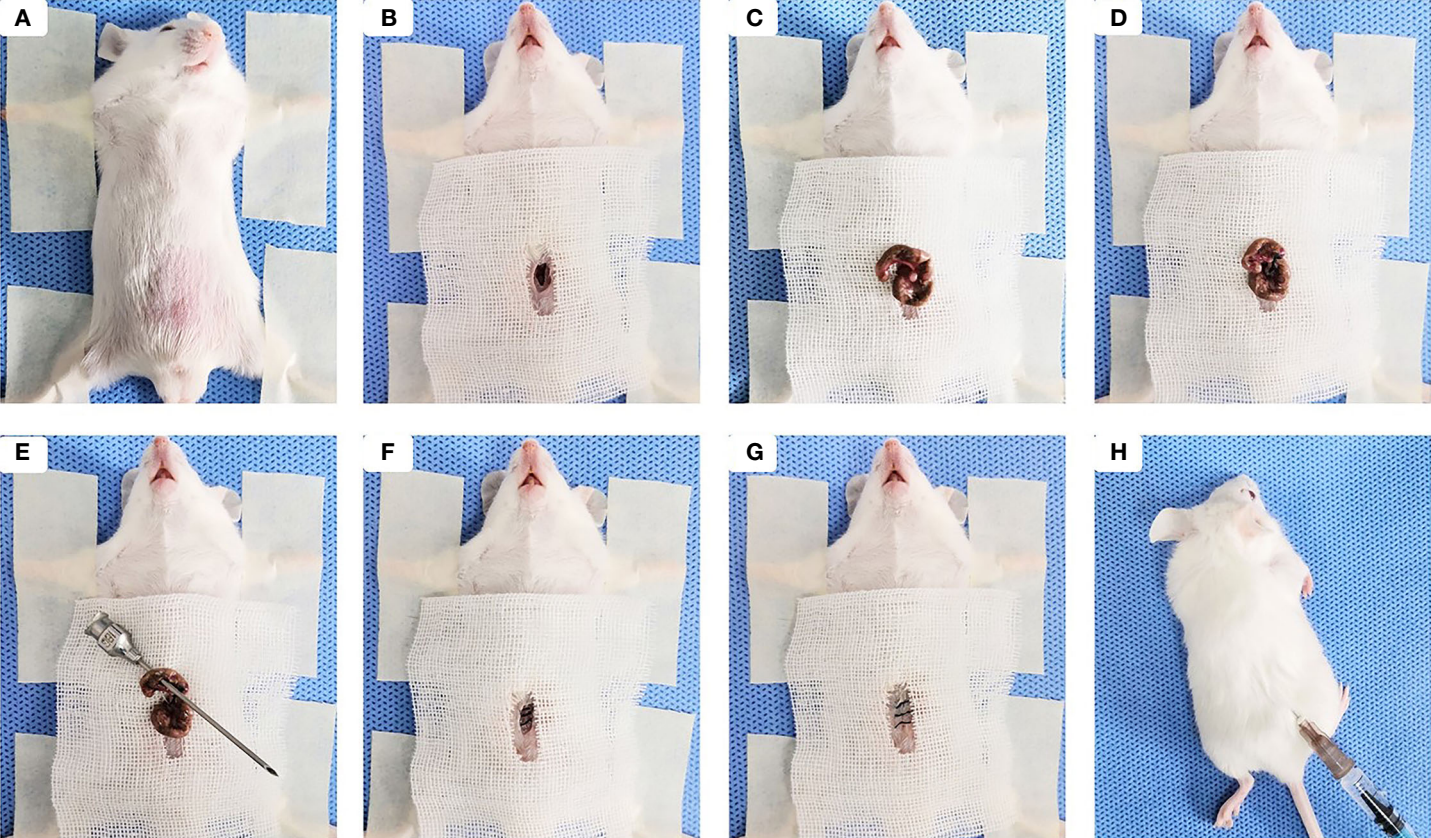
Sepsis-induced ALI was established by the cecal ligation and puncture (CLP). **(A)** The mouse was placed in a supine position under anesthesia, then the abdominal area was shaved, cleaned, and disinfected. **(B)** Skin midline incision was made. **(C)** The cecum was isolated. **(D)** The cecum was ligated tightly with silk at 1.5 cm from the tip. **(E)** Cecal puncture (a through-and-through) was made after cecum ligation. **(F)** The cecum was returned back to the abdominal cavity and muscle layer was closed. **(G)** The skin incision was disinfected after suturing. **(H)** 200 µl of sterile saline was injected sub-dermally to each mouse.

Sepsis model was established on the basis of the cecal ligation and puncture (CLP) (Rittirsch et al., 2009). Briefly, Male BALB/c mice were fasted for 12 h with drinking water only. A 0.8-cm midline incision was made for each mouse to expose the cecum under anesthesia with intraperitoneal injection of 4% chloral hydrate (0.2 ml/20 g) (**Figures 1A, B**). The cecum was isolated and ligated tightly with a 3.0 silk at 1.5 cm from the tip (**Figures 1C, D**). A through-and-through puncture was made on the cecum using an 18-gauge needle and a small amount of feces was extruded (**Figure 1E**). The cecum was returned back to the abdominal cavity and each opened layer was closed with 4.0 silk sutures (**Figures 1F, G**). Following CLP, 200 µl of sterile saline was injected sub-dermally to each mouse for fluid resuscitation (**Figure 1H**). The same laparotomy was operated for sham control group without cecum ligation and puncture.

### Treatment of Sepsis With *Ts*-AES

BABL/c mice were randomly divided into 4 groups with 16 mice each: (i) sham-operated (mice underwent the same procedure, except for ligation and puncture of the cecum) with PBS (Sham+PBS), (ii) CLP with PBS (CLP+PBS), (iii) CLP treated with *Ts*-AES (CLP+AES), (iv) Sham-operated treated with *Ts*-AES (Sham+AES). CLP+PBS and CLP+AES groups were CLP operated and then treated intraperitoneally with PBS or 15 μg of *Ts*-AES in a total volume of 200 μl 30 min after surgery, respectively. Sham+PBS and Sham+AES groups were performed sham surgery without ligation and puncture of the cecum and given with the same amount of PBS or *Ts*-AES after surgery, respectively. Six mice in each group were sacrificed 12 h after surgery and treatment for measuring the level of inflammatory cytokines in blood and lung and pathological change in lung tissue. The remaining 10 mice were observed for general physical conditions and survival rate for 72 h. The survival rates were determined using Kaplan-Meier method.

### Detection of Cytokines in Sera Using Enzyme-Linked Immunosorbent Assay (ELISA)

Six mice from each group were anesthetized with 4% chloral hydrate and blood samples were collected from the fundus venous plexus 12 h after surgery. The sera were collected from the blood samples by centrifuging at 4000 rpm for 15 min. The serological levels of the pro-inflammatory cytokines (TNF-α, IL-6, IL-1β) and regulatory cytokines (IL-10 and TGF-β) were determined using LEGEND MAX™ ELISA kits (Dakewe Biotech, Beijing, China) according to the manufacturer’s instructions.

### Lung Collection for Pathological Observation

After blood samples were collected 12 hours after surgery, the six mice from each group were euthanized with cervical dislocation and the lung tissues were collected by postmortem bilateral thoracotomy. The left lungs were frozen at -80°C until use for quantitative real time PCR *(*qRT-PCR) and western blot analysis. The right lungs were collected for the determination of lung wet/dry weight ratio and histopathological changes. Superior and middle lobes of right lung were collected and the wet weight of the tissue was weighed immediately, then the lung tissues were dried out by baking in a hot air oven at 70°C for 48 h and weighed. The lung wet/dry weight ratio was calculated to assess tissue edema. The inferior and postcaval lobes of right lung samples were fixed in 4% paraformaldehyde neutral buffer solution and cut into 5 μm sections and stained with hematoxylin and eosin (HE). The stained sections were observed under microscope (Olympus, Tokyo, Japan). The histological lung injury was scored as 0-4 based on 4 criteria: 1) alveolar hemorrhage and congestion; 2) alveolar edema; 3) alveolar or vascular wall neutrophil infiltration or aggregation; and 4) alveolar septum thickening, according to lesion severity as shown in **Table 1** (Meng et al., 2018; Shao et al., 2019).

**Table 1:** Lung injury score parameters.

Index	Alveolar hemorrhage and congestion	Alveolar edema	Alveolar or vascular wall neutrophil infiltration or aggregation	Alveolar septum thickening
0	None or very mild lesions			
1	Mild lesions (<25% lung involvement)			
2	Moderate lesions (25–50% lung involvement)			
3	Severe lesions (50–75% lung involvement)			
4	Very severe lesions (>75% lung involvement)			

### Quantitative Real Time PCR (qRT-PCR)

Total RNA was extracted from left lung samples using TRIzol™ Reagent (Invitrogen, Carlsbad, CA, USA) and reversely transcribed to cDNA by using the Superscript First Strand cDNA Synthesis Kit (Thermo Fisher Scientific Inc., Waltham, MA, USA) according to the manufacturer’s instructions. A quantitative analysis of the relative mRNA expression of different cytokines (TNF-α, IL-6, IL-1β, IL-10 and TGF-β), HMGB1, TLR2 and MyD88 in murine lung tissues were determined in triplicate using SYBR Green Super Mix Kit (Takara Bio Inc., Tokyo, Japan) on a Roche LightCycler^®^ 96 real-time PCR system (Roche Molecular Systems, Inc., USA). The relative mRNA expression was calculated with the comparative △Cq method using the formula 2^−△△Cq^ compared to GAPGH housekeeper gene control. The primers listed in **Table 2** were designed and synthesized by Sangon Biotech (Shanghai, China) for testing each gene expression.

**Table 2:** The primers of qRT-PCR.

Primer	Forward	Reverse
TNF-α	ACGGCATGGATCTCAAAGAC	GTGGGTGAGGAGCACGTAGT
IL-6	CCGGAGAGGAGACTTCACAG	TCCACGATTTCCCAGAGAAC
IL-1β	TCTTTGAAGAAGAGCCCATCC	CTAATGGGAACGTCACACAC
TGF-β	CTGGATACCAACTACTGCTTCAG	TTGGTTGTAGAGGGCAAGGACCT
IL-10	CCAAGCCTTATCGGAAATGA	TTTTCACAGGGGAGAAATCG
TLR2	TGCAAGTACGAACTGGACTTCT	CCAGGTAGGTCTTGGTGTTCATT
HMGB1	GGCGAGCATCCTGGCTTATC	AGGCAGCAATATCCTTCTCATAC
MyD88	ACTGGCCTGAGCAACTAGGA	CGTGCCACTACCTGTAGCAA
GAPDH	ACCCAGAAGACTGTGGATGG	CACATTGGGGGTAGGAACAC

### Detection of HMGB1, TLR2, and MyD88 Expression in Lung Tissue by Western Blotting

The protein of left lung samples was isolated by dissolving tissue with RIPA buffer containing 0.1% phenylmethylsulfonyl fluoride (PMSF) and centrifuged at 12000 rpm for 20 min at 4°C. The proteins were separated using 12% SDS-PAGE and transferred onto polyvinylidene difluoride (PVDF) membranes. The membranes were blocked with 5% nonfat dry milk in Tris-buffered saline containing 0.05% Tween-20 (TBST), and then incubated overnight at 4°C with following primary antibodies: anti-β-actin (1:2000), anti-HMGB1(1:1000) anti-MyD88(1:1000), (Cell Signaling Technology, Danvers, Massachusetts, USA) and anti-TLR2(1:1500) (Abcam, Cambridge, UK), respectively. Goat anti-mouse IgG was used as secondary antibody (Merck Millipore, Basilica, Massachusetts, USA) at 1:8,000 dilution for 1 h at 37°C. Immunoreactive protein bands were visualized using a Bio-Rad ChemiDoc XRS+ Chemiluminescence imaging system (Bio-Rad, Hercules, CA, USA). Semi-quantitatively analyses on developed bands were performed by ImageJ software.

### Flow Cytometry

Spleen was collected from each euthanized mouse 12 hours after surgery and treatment, the single cell suspension was prepared in PBS containing 2% FBS for staining. The cell surfaces were blocked with rat anti-mouse CD16/CD32 antibody for 15 min at 4°C, and then incubated with PE*-*Cy7*-*anti-CD3Ɛ, FITC-anti-CD4 and APC-anti-CD25 antibodies (Biolegend, London, United Kingdom) for surface marker staining. After washing, fixation, permeabilization and a second blocking, the intracellular labeling of Foxp3 protein was performed by incubating the cells with PE-anti-Foxp3 antibody for 30 min on ice in the dark. The isotype-matched immunoglobulins (Biolegend, London, United Kingdom) and FMO were used as control for non-specific staining as baseline. The cells were washed twice with fluorescence-activated cell sorting staining buffer and analyzed by DxP Athena™ flow cytometer (CYTEK, USA). All the results were analyzed using FlowJo^R^ 7.6 software (Treestar, Ashland, OR, USA).

### Statistical Analysis

All data were presented as the mean ± standard deviation (SD), and statistical analyses were performed using GraphPad Prism 5.0 software (GraphPad Inc., La Jolla, CA, USA). Comparison of the same parameters in multiple datasets or more than two groups was done using one-way analysis of variance (ANOVA). The difference in survival rates among four groups was compared using Kaplan-Meier survival analysis. *P* < 0.05 was considered as statistically significant.

## Results

### *Ts*-AES Improved the Survival Rate of Septic Mice

**Figure 2:**
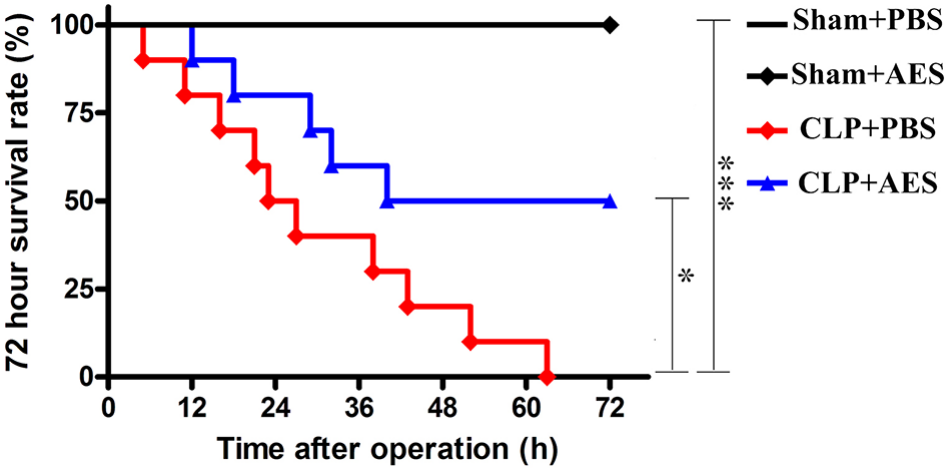
*Ts*-AES treatment improved the survival rate of mice with CLP-induced sepsis. After CLP or sham operation, mice were injected intraperitoneally with *Ts*-AES or PBS. The survival rate was determined using Kaplan Meier method and compared by log-rank test (n = 10 mice per group). *P < 0.05, ***P < 0.001.

*Ts-*AES was used to treat sepsis in a CLP-induced sepsis mouse model, to determine whether it improves the survival rate of septic mice. As shown in **Figure 2**, all mice died 72 h after CLP operation treated with PBS only (CLP+PBS). However, the 72 hours survival rate for septic mice treated intraperitoneally with 15 μg of *Ts*-AES (CLP+AES) increased to 50% with significant difference compared to CLP group without treatment (CLP+PBS) (*P*<0.05). All mice in sham groups with or without *Ts*-AES treatment survived for 72 h period. This result suggests that treatment with *Ts*-AES significantly improves the survival rate of mice with sepsis.

### *Ts*-AES Reduced Sepsis-Caused Acute Lung Injury (ALI)

**Figure 3:**
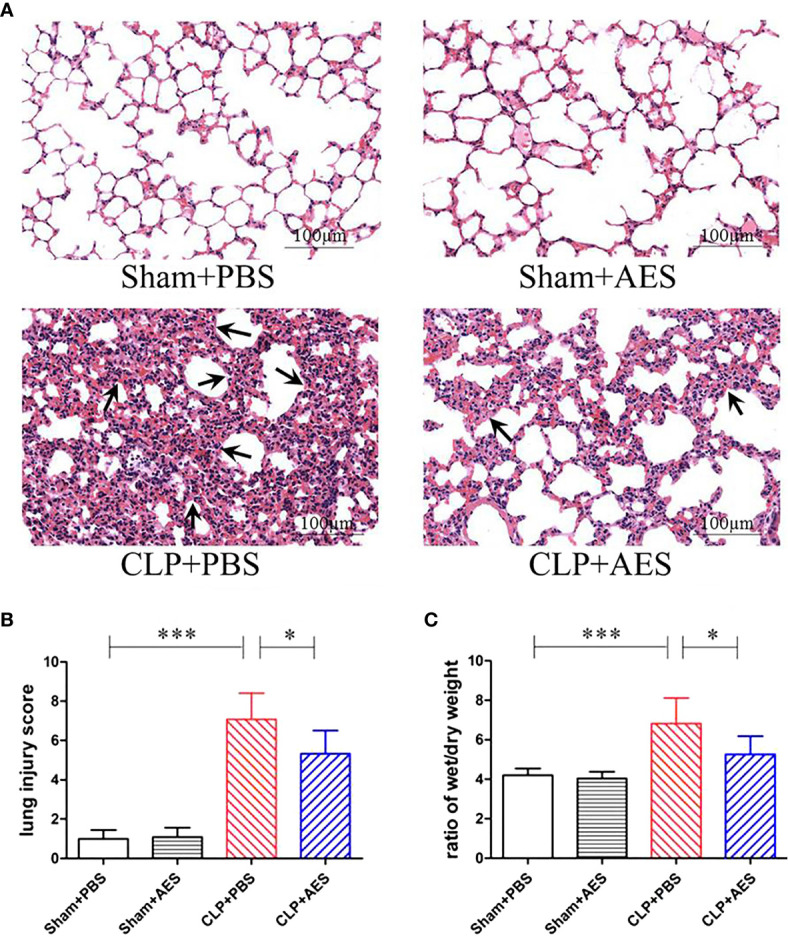
Treatment with *Ts*-AES reduced ALI caused by CLP-induced sepsis. **(A)** Representative lung tissue sections in different groups exhibiting alveolar structural disruption and alveolar septum thickening with the inflammatory cell infiltration in septic mice (CLP+PBS). However, the lung tissue pathological changes caused by sepsis were significantly attenuated in CLP mice treated with *Ts*-AES (CLP+AES) (×200; Scale-bars: 100 μm). The arrows indicate thickened alveolar septum. **(B)** The improved lung injury score after being treated with *Ts*-AES (CLP+AES) compared with CLP+PBS. **(C)** Pulmonary edema assessed by wet/dry weight ratio of lung tissue was significantly reduced in CLP mice treated with *Ts*-AES compared with CLP mice without treatment. The results are shown as the means ± SD for each group (n = 6). *P < 0.05, ***P < 0.001.

The lung tissue structure and pathological changes of mice 12 h after CLP surgery and treatment were determined by histochemical staining of lung tissue sections. The results showed that Sham+PBS group and Sham+AES group had no evidence of inflammation with normal arrangement of the lung structure. However, the lung tissue in CLP+PBS group showed alveolar congestion, structural disruption and thickened alveolar septum with the inflammatory cell infiltration, showing typical pathology of acute lung injury (ALI) (**Figure 3A**) with high lung injury scores and high ratio of wet/dry weight (**Figures 3B, C**). After treatment with *Ts*-AES, the lung injury caused by sepsis was significantly mitigated with significantly reduced inflammatory cell infiltration, lung injury scores compared to mice with CLP-induced sepsis without treatment (CLP+PBS) (**Figures 3A, B**). The sepsis inflammation caused pulmonary edema was also significantly reduced in CLP group treated with *Ts*-AES compared to CLP group treated with PBS only based on the reduced ratio of wet/dry weight (*P*<0.05) (**Figure 3C**). These results suggest that treatment with *Ts*-AES significantly attenuates the lung tissue pathology caused by sepsis and reduces the water content of lung tissue and pulmonary edema in septic ALI mice caused by inflammatory congestion and infiltration.

### *Ts*-AES Inhibited Pro-inflammatory Cytokines and Induced IL-10 and TGF-β in Septic Mice

**Figure 4:**
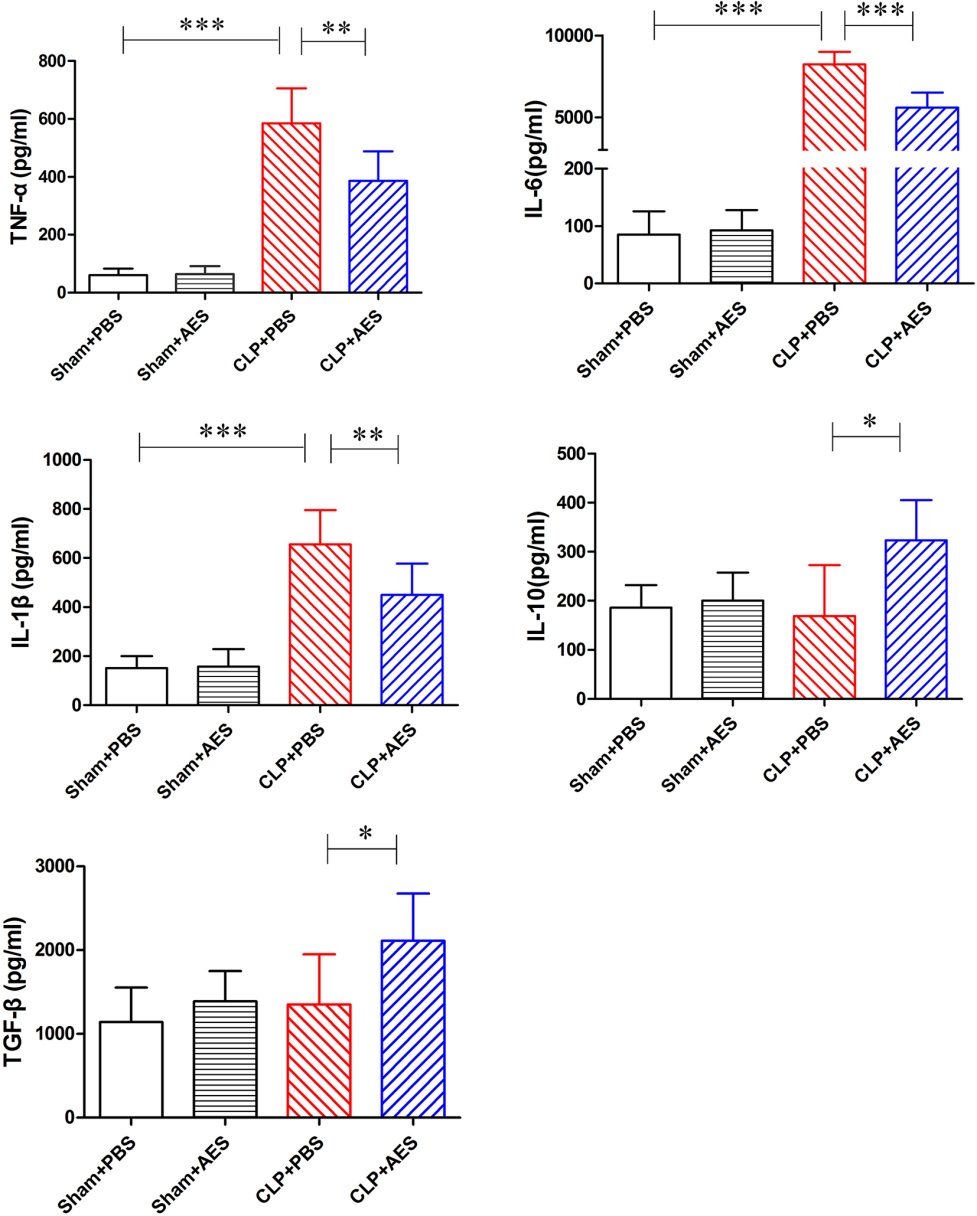
Treatment with *Ts*-AES reduced the inflammatory cytokines TNF-α, IL-6, IL-1β and stimulated regulatory cytokines IL-10 and TGF-β in sera of CLP-induced septic mice. The levels of these cytokines in sera of mice were measured by ELISA 12 h after the surgery. The results were shown as the mean ± SD for each group (n = 6). *P < 0.05, **P < 0.01, ***P < 0.001.

Sepsis was characterized by surged pro-inflammatory cytokines in the blood circulation (Hübner et al., 2013) which induced acute lung injury. We examined the serological levels of some pro-inflammatory cytokines in mice of each group and revealed that TNF-α, IL-6 and IL-1β were significantly increased in septic mice 12 h after CLP operation (CLP+PBS). However, treatment with *Ts*-AES significantly reduced their serological levels of TNF-α, IL-6 and IL-1β (CLP+AES) compared to those without treatment (CLP+PBS) (**Figure 4**). We also found that treatment with *Ts*-AES stimulated the secretion of IL-10 and TGF-β in sera of septic mice (CLP+AES) compared to CLP group without treatment (CLP+PBS). Treatment with *Ts*-AES had no effect on the serological levels of pro-inflammatory cytokines (TNF-α, IL-6, IL-1β) or regulatory cytokines (IL-10 and TGF-β) in sham surgery groups with or without *Ts*-AES treatment (Sham+AES or Sham+PBS). These data indicated that *Ts*-AES inhibited inflammatory cytokines in septic mice associated with increased serological levels of regulatory cytokines IL-10 and TGF-β.

**Figure 5:**
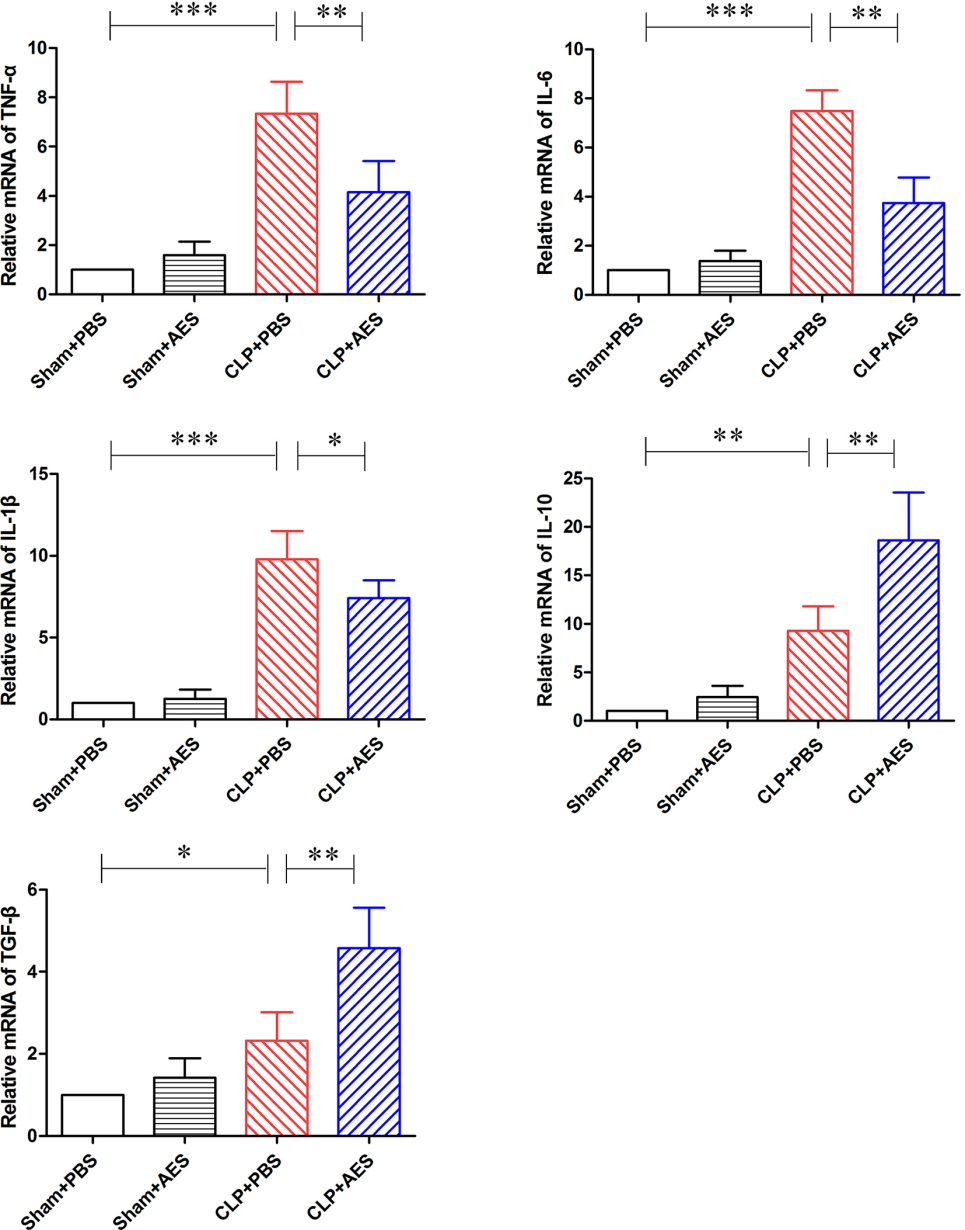
Treatment with *Ts*-AES reduced the mRNA expression levels of pro-inflammatory cytokine (TNF-α, IL-6 and IL-1β) and increased the mRNA expression levels of regulatory cytokine (IL-10 and TGF-β) in lung tissues of septic mice. The results were shown as the means ± SD for each group (n=6). *P < 0.05, **P < 0.01, ***P < 0.001.

The similar effects of *Ts*-AES treatment on these cytokines were also identified in lung tissue by measuring their mRNA levels. The qRT-PCR results showed that the mRNA expression levels of pro-inflammatory cytokines TNF-α, IL-6 and IL-1β were significantly reduced in lung tissue of septic mice treated with *Ts*-AES (CLP+AES) compared to the septic mice treated with PBS only (CLP+PBS). The reduced mRNA expression levels of pro-inflammatory cytokines were correlated with the increased regulatory cytokines IL-10 and TGF-β mRNA expression levels in lung tissues of septic mice treated with *Ts*-AES (CLP+AES) compared with the CLP group without treatment (CLP+PBS) (**Figure 5**). However, there was no change of mRNA levels for either pro-inflammatory cytokines or regulatory cytokines in lung tissue of sham surgery mice with or without *Ts*-AES treatment. The results suggest that treatment with *Ts*-AES directly inhibits pro-inflammatory cytokine expression and stimulates the expression of regulatory cytokines that is correlated with the improvement of acute lung injury caused by CLP-induced sepsis.

### Treatment With *Ts*-AES Stimulated Regulatory T-Cells (Tregs)

**Figure 6:**
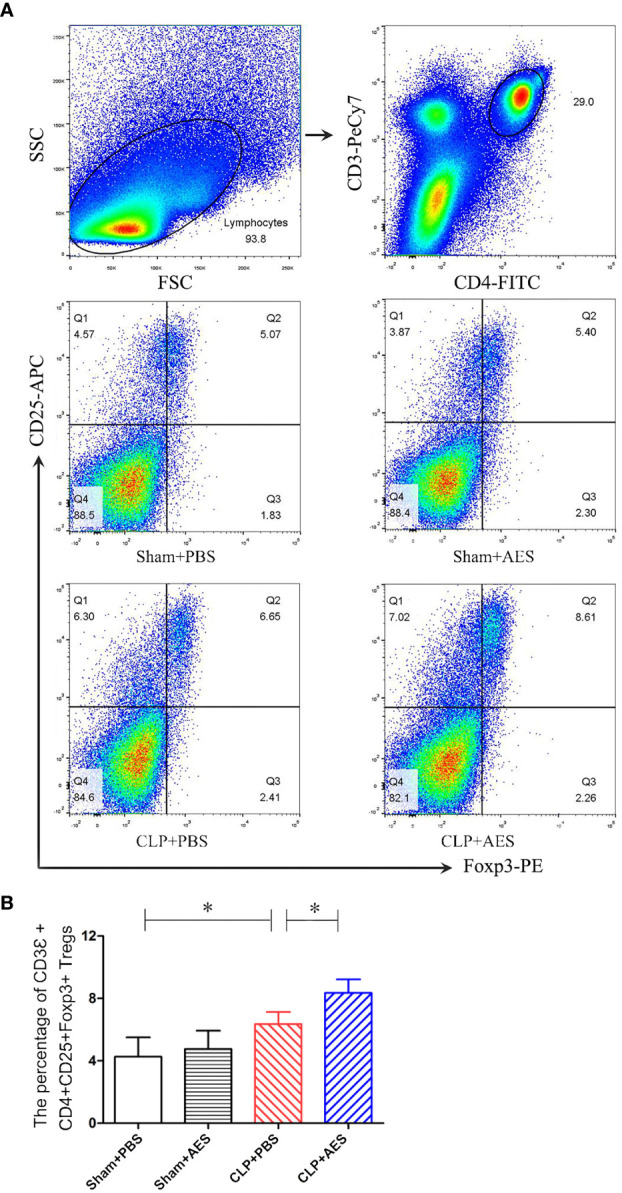
Treatment with *Ts-*AES increased percentage of CD3Ɛ^+^CD4^+^CD25^+^Foxp3^+^ Tregs in the spleen lymphocytes of mice with CLP-induced sepsis. The percentage of CD3Ɛ^+^CD4^+^CD25^+^Foxp3^+^ Tregs in the splenocytes of mice was gated and counted. **(A)** Representative three-dimension scatter diagrams of CD3Ɛ^+^CD4^+^CD25^+^Foxp3^+^ Tregs in the splenocytes of mice in each group. **(B)** The percentage of CD3Ɛ^+^CD4^+^CD25^+^Foxp3^+^ Tregs in the splenocytes of mice in each group. The results were shown as the means ± SD for each group (n = 6). *P < 0.05.

Many studied have demonstrated that Tregs play a key role in modulating the inflammatory responses to relieve the sepsis-related immunopathology (Xu et al., 2020). To further examine whether the reduced inflammation and acute lung injury in septic mice treated with *Ts*-AES was associated with the activation of Treg response, the Tregs with surface expressions of CD3Ɛ, CD4 and CD25 and the intracellular expression of Foxp3 were examined in lymphocytes isolated from the spleens of mice using flow cytometry. As shown in **Figure 6**, Treatment with *Ts*-AES induced CD3Ɛ^+^CD4^+^CD25^+^Foxp3^+^ Tregs in splenocytes from septic mice (CLP+AES) compared with those in septic mice without treatment (CLP+PBS). The severe infection itself also stimulated CD3Ɛ^+^CD4^+^CD25^+^Foxp3^+^ Tregs in the spleen cells from septic mice (CLP+PBS), but the increase of Tregs is more significant after treatment with *Ts*-AES. The results further suggest that *Ts*-AES modulate host immune system by stimulating regulatory T cell response. The results are correlated with the reduced pro-inflammatory cytokines and elevated regulator cytokines IL-10 and TGF-β in sera and lung tissues measured above.

### *Ts*-AES Alleviated Sepsis-Induced ALI *via* HMGB1/TLR2/MyD88 Signaling Pathway

**Figure 7:**
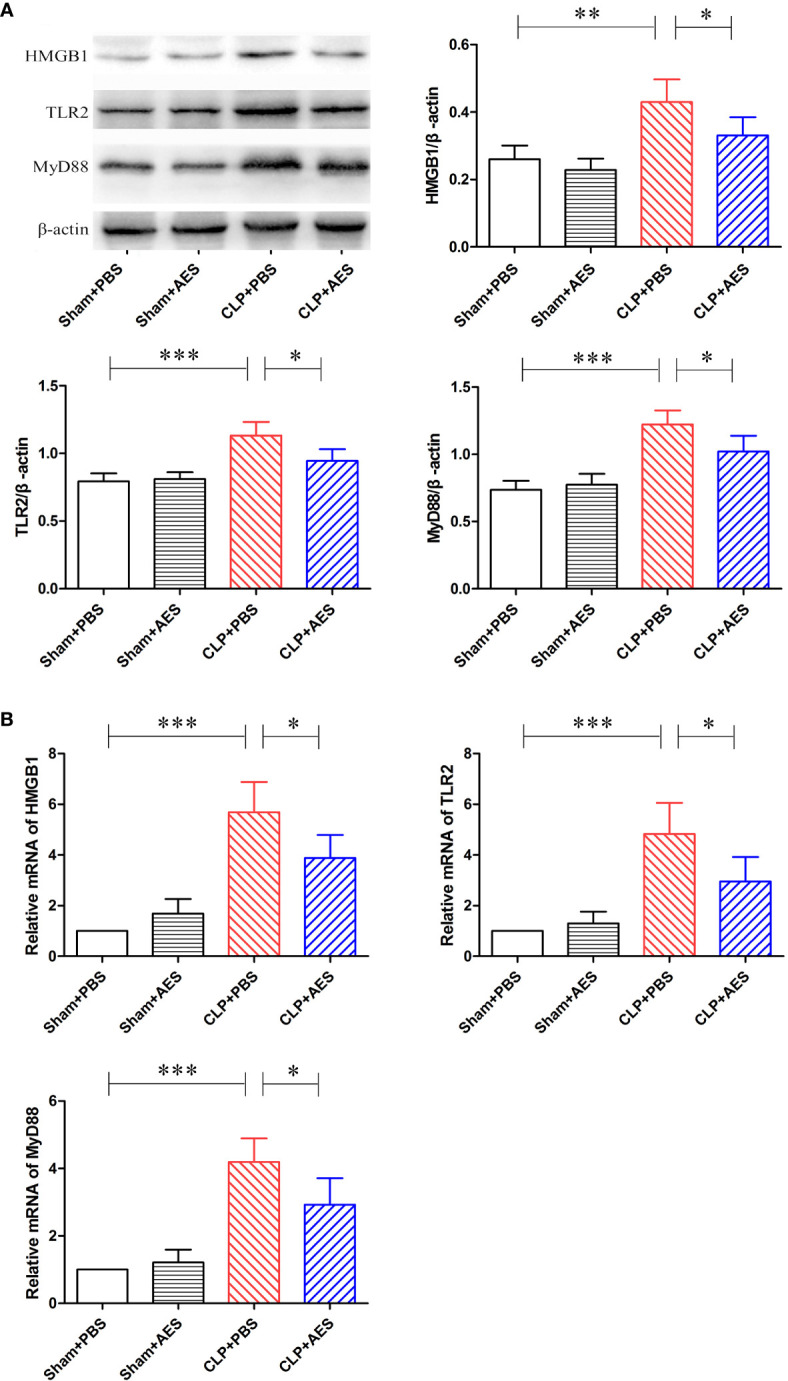
Treatment with *Ts*-AES reduced the protein expression levels of HMGB1, TLR2 and MyD88, β-actin was measured as a control **(A)**, and the mRNA expression levels of HMGB1, TLR2 and MyD88 in lung tissues of septic mice **(B)**. The results were shown as the means ± SD for each group (n = 6). *P < 0.05, **P < 0.01, ***P < 0.001.

HMGB1 is a regulatory protein in nucleus related to activate the family of Toll-like receptors (TLRs) (Park et al., 2004; Yu et al., 2006) and TLR/MyD88 is an important signaling pathway involved in the inflammation (Kumar, 2020). To determine whether the HMGB1/TLR2/MyD88 signaling pathway is involved in the therapeutic effect of *Ts*-AES on sepsis-induced ALI, we evaluated protein and mRNA expression levels of HMGB1, TLR2 and MyD88 in lung tissue 12 h after CLP surgery. The elevated levels of HMGB1, TLR2 and MyD88 proteins were observed in lung tissue of mice 12 h after CLP operation in CLP+PBS group compared with the mice in sham groups with or without treatment of *Ts*-AES. Treatment with *Ts*-AES significantly reduced the protein expression levels of HMGB1, TLR2 and MyD88 in lung tissues of septic mice (CLP+AES) compared with mice without *Ts*-AES treatment (CLP+PBS) (**Figure 7A**). However, there was no significant difference in protein expression of HMGB1, TLR2 and MyD88 in lung tissue between Sham+PBS group and Sham+AES group. The mRNA expression levels of HMGB1, TLR2 and MyD88 detected in lung tissue showed a similar pattern to the proteins measured in lung tissue (**Figure 7B**). These results indicate that *Ts*-AES could alleviate sepsis-induced acute lung injury possibly through inhibiting HMGB1, TLR2 and MyD88 pathway.

## Discussion

Sepsis is overwhelming immune responses upon systemic infection that trigger inflammatory storm throughout the body to cause multiple organ damage. The lung is particularly susceptible to acute injury during sepsis. Studies have found that more than 50% of patients with sepsis develop ALI or acute respiratory distress syndrome (ARDS) confirmed both in animal and clinical observations (Carvelli et al., 2019). In this study, we confirmed septic ALI in a CLP-induced sepsis mouse model characterized by structure damage and inflammatory cell infiltration, pulmonary edema in lung tissue accompanied by the increased levels of pro-inflammatory cytokines, which closely mimicked the pathophysiological characteristics of acute lung injury observed in clinical patients.

The ES products of helminth have been regarded as key molecules secreted by helminth in regulating host inflammation, cell apoptosis, protein degradation and antigen presentation (Crowe et al., 2017; Smallwood et al., 2017; Pan et al., 2018; Sun et al., 2019a). Previous studies have determined that *Ts*-AES enabled to induce strong Tregs responses, characterized by increased CD4^+^CD25^+^Foxp3^+^ and CD4^+^CD25^−^Foxp3^+^ Treg cells accompanied by high levels of IL-10 and TGF-β (Sun et al., 2019b). Treatment with *Ts*-AES significantly alleviated Th1-dominated colonic inflammation and pathology in DSS-induced colitis in mice (Yang et al., 2014). In this study, we would like to determine whether *Ts*-AES modulate the immune response against systemic sepsis and protect mice from septic acute lung injury.

The early phase of sepsis is characterized by excessive inflammation with the manifestation of systemically boosted production of pro-inflammatory cytokines, including TNF-α, IL-6 and IL-1β (Sackett et al., 2019). The pro-inflammatory cytokine storm can induce lung endothelial cell activation, leukocyte migration, and capillary leakage that result in lung edema to further hinder alveolar cell perfusion and oxygen exchange, thus causing ALI (Chaudhry et al., 2013). In this study, we observed that 12 hours after CLP operation, the mRNA expression levels of pro-inflammatory cytokines (TNF-α, IL-6 and IL-1β) in lung tissues were significantly increased and the structure of lung tissue was damaged including alveolar congestion and thickened alveolar septum with the inflammatory cell infiltration, which is consistent with the increased levels of TNF-α, IL-6 and IL-1β in sera. Treatment with 15 µg of *Ts*-AES significantly improved survival rate of mice with CLP-induced sepsis up to 50% at 72-hour time point after CLP operation compared with septic mice without treatment that all died at the same time point (*P*<0.05). Histochemical staining of lung tissue identified that the pathology of sepsis-caused ALI was significantly reduced in mice treated with *Ts*-AES, with reduced inflammatory cell infiltration and congestion, reduced wet/dry weight ratio of lung tissue, less lung tissue damage and structural disruption, and improved lung injury scores, compared to lung tissues of septic mice without treatment (**Figures 3A–C**). The reduced pathology of sepsis-caused ALI was correlated with reduced levels of pro-inflammation cytokines such as TNF-α, IL-6 and IL-1β in blood and their mRNA transcriptional level directly in lung tissue.

As we know, Treg cells play a key role in the induction of immune homeostasis and tolerance mainly through the secretion of IL-10 and TGF-β to exert regulatory influence on the immune system (Smallwood et al., 2017). In this study, we identified that the proportion of CD3Ɛ^+^CD4^+^CD25^+^Foxp3^+^ Tregs in the spleen cells were increased in septic mice upon the treatment of *Ts*-AES after CLP operation, which is correlated with the increased levels of IL-10 and TGF-β and decreased levels of pro-inflammatory cytokines (TNF-α, IL-6 and IL-1β) in sera and lung tissue, indicating that *Ts*-AES act as an inhibitory immunomodulator in the case of excessive inflammatory infection, possibly through stimulating Tregs and Treg cell-secreted IL-10 and TGF-β to reduce sepsis-induced acute lung injury and therefore improve the survival rate of mice with septic ALI. We observe in this study that septic infection itself also increased Tregs (**Figure 6B**), however, it has been suggested the increased Tregs in inflammatory diseases, such as colitis, sepsis, the function of Tregs is typically impaired in these diseases, and the impaired Tregs are usually unable to restrict the excessive inflammation (Yang et al., 2014; Xu et al., 2020).

During the early onset of sepsis, the innate immune system is activated through toll-like receptors (TLRs) through pathogen-associated molecular patterns (PAMPs) signaling pathway. Activation of TLRs induces the rapid release of early pro-damage signals, including damage-associated molecular patterns (DAMPs) that are referred to as alarmins, into the circulation. High Mobility Group Box 1 protein (HMGB1) is a major alarmin which is passively released from injured or necrotic cells in sepsis (Tian et al., 2020). HMGB1 is typically found in the nucleus of many cells including immune, endothelial and epithelial cells where it is bound to DNA. After being stimulated by bacterial endotoxin or pro-inflammatory cytokines in the case of sepsis, HMGB1 was acetylated and released as a cytokine mediator of inflammation through receptors for advanced glycation end products (RAGEs) and TLRs (TLR2 and TLR4) to stimulate excessive release of pro-inflammatory cytokines (Park et al., 2004; Yu et al., 2006; Kang et al., 2010; Andersson and Tracey, 2011). In this study we found that the protein expression levels and the mRNA expression levels of HMGB1, TLR2 and MyD88 in lung tissue are significantly increased associated with the high levels of pro-inflammatory cytokines in sera and lung tissues 12 hours after CLP operation. After being treated with *Ts*-AES, protein expression levels and the mRNA expression levels of HMGB1, TLR2 and MyD88 in lung tissues were significantly decreased. Our results further confirm that severe infection of bacteria in sepsis stimulates inflammatory immune responses through HMGB1/TLR2/MyD88 activation signal pathway as shown for other helminth-derived proteins (Li et al., 2017; Ilic et al., 2018; Gao et al., 2020).

In this study we have demonstrated *Ts*-AES play an important role in modulating host immune response possibly through stimulating Tregs response and promoting secretion of IL-10 and TGF-β, thereby suppressing the production of pro-inflammatory cytokines. The immunomodulation of *Ts*-AES is taken effect possibly through inhibiting the HMGB1/TLR2/MyD88 activation signal pathway. However, there are still a lot that are not clear and need to be further explored, for example, what the effective cells to which HMGB1/TLR2/MyD88 activation signal pathway regulates, macrophages, dendritic cells or other immune cells? It needs to be further investigated.

There are some concerns for using *Ts*-AES as therapeutic reagents to reduce systemic infection-caused lung and other organ injury, one of the concerns is that administration of *Ts*-AES could induce antibody response that may reduce the efficacy of *Ts*-AES. However, the administration of *Ts*-AES was through intraperitoneal injection without using any adjuvant in this study. As we know, intraperitoneal administration without adjuvant within limited time period could not effectively induce much antibody response. However, it would be expected to reduce efficacy if *Ts*-AES are used repeatedly or for a long time period due to the potential antibody production that would neutralize the efficacy of *Ts*-AES. Another concern is for the complicated composition of *Ts*-AES. *Ts*-AES is a complex pool of various molecules secreted or excreted by worms, it is difficult to be accepted by using this complex as therapeutic reagents because of the possible side-effects or immune interference. It is also difficult to make large-scale manufacture of *Ts*-AES that limits its use in clinical treatment. It is important to identify the effective components in *Ts*-AES that are involved in the regulatory activation of immune cells. Shotgun LC-MS/MS approach identified more than 280 protein components in *Ts*-AES with 4 proteins having potential regulatory functions including cysteine protease inhibitor, serine protease, 53 kDa excretory-secretory protein, and glutathione-S-transferase (Yang et al., 2017; Wang Z. et al., 2020). Recent studies have confirmed that recombinant *T. spiralis* cysteine protease inhibitor relieved TNBS-induced experimental inflammatory bowel disease by inducing Th2-type immune response and balanced the Th1-type immune response induced by TNBS administration (Xu et al., 2019). Serine protease of *Ts*-AES alleviated TNBS-induced colitis by increasing the population of Th2 and Treg cells (Pang et al., 2020). Recombinant *T. spiralis* 53-kDa excretory-secretory protein exhibited anti-inflammatory properties and rescued mice from LPS-induced damage of endotoxemia (Chen et al., 2016). Recombinant *T. spiralis* glutathione-S-transferase decreased the LPS-induced elevated level of pro-inflammatory cytokines of dendritic cells and enhanced the level of regulatory cytokines IL-10 and TGF-β (Jin et al., 2019b). The identification of the immunomodulatory molecules in *Ts*-AES will make it possible to develop effective therapeutic drugs for inflammatory and immune diseases. The related research on *Ts*-AES is under investigation in our laboratory.

## Conclusions

In this study we have presented results demonstrating that *Ts*-AES strongly alleviate excessive inflammation *via* stimulating Tregs response and inhibiting the HMGB1/TLR2/MyD88 signal pathway, and protect mice from ALI induced by sepsis. Therefore, *Ts*-AES could be considered as a potential therapeutic agent for the treatment of severe infection or other inflammatory/auto immune diseases.

## Data Availability Statement

The original contributions presented in the study are included in the article/supplementary material. Further inquiries can be directed to the corresponding author.

## Ethics Statement

The animal study was reviewed and approved by Animal Care and Use Committee of Bengbu Medical College (approval no: LAEC-2014-039).

## Author Contributions

XY, HL, and BZ conceived and designed the study. HL, DQ, HY, XW, LW, YS, and WX performed the experiments. HL, YY, and LC analyzed the data. HL wrote the manuscript. BZ and XY critically revised the manuscript. All authors contributed to the article and approved the submitted version.

## Funding

This study was supported by the Science Foundation of Anhui Province (no. 2008085MH260, gxbjZD15); Program of Natural Science Foundation of the Anhui Higher Education Institutions (no. KJ2020A0554, KJ2020A0572); 512 Talents Development Program of Bengbu Medical College (no. by51201205, by51201306); the innovation and entrepreneurship training program for college students (no. 201910367025, S202010367040).

## Conflict of Interest

The authors declare that the research was conducted in the absence of any commercial or financial relationships that could be construed as a potential conflict of interest.
